# Case of 7p22.1 Microduplication Detected by Whole Genome Microarray (REVEAL) in Workup of Child Diagnosed with Autism

**DOI:** 10.1155/2015/212436

**Published:** 2015-03-29

**Authors:** Veronica Goitia, Marcial Oquendo, Robert Stratton

**Affiliations:** ^1^Department of Pediatrics, Driscoll Children's Hospital, Corpus Christi, TX 78411, USA; ^2^Department of Medical Genetics, Driscoll Children's Hospital, Corpus Christi, TX 78411, USA

## Abstract

*Introduction*. More than 60 cases of 7p22 duplications and deletions have been reported with over 16 of them occurring without concomitant chromosomal abnormalities.* Patient and Methods*. We report a 29-month-old male diagnosed with autism. Whole genome chromosome SNP microarray (REVEAL) demonstrated a 1.3 Mb interstitial duplication of 7p22.1 ->p22.1 arr 7p22.1 (5,436,367–6,762,394), the second smallest interstitial 7p duplication reported to date. This interval included 14 OMIM annotated genes (*FBXL18, ACTB, FSCN1, RNF216, OCM, EIF2AK1, AIMP2, PMS2, CYTH3, RAC1, DAGLB, KDELR2, GRID2IP*,* and ZNF12*).* Results*. Our patient presented features similar to previously reported cases with 7p22 duplication, including brachycephaly, prominent ears, cryptorchidism, speech delay, poor eye contact, and outburst of aggressive behavior with autism-like features. Among the genes located in the duplicated segment,* ACTB* gene has been proposed as a candidate gene for the alteration of craniofacial development. Overexpression of* RNF216L* has been linked to autism. FSCN1 may play a role in neurodevelopmental disease.* Conclusion*. Characterization of a possible 7p22.1 Duplication Syndrome has yet to be made. Recognition of the clinical spectrum in patients with a smaller duplication of 7p should prove valuable for determining the minimal critical region, helping delineate a better prediction of outcome and genetic counseling

## 1. Introduction

More than 60 cases of 7p22 duplications and deletions have been reported in [1] with over 16 of them occurring without concomitant chromosomal abnormalities [2]. Several cases of de novo 7p duplications have been reported in recent years [2–4]; however, familial cases due to malsegregation of a parental balanced translocation or abnormal recombination caused by a parental inversion seem to be the most common cause of 7p duplications [5, 6]. These patients often include findings such as developmental delay, intellectual disability, behavioral problems, abnormal speech development, autism spectrum disorder (ASD), hypotonia, craniofacial dysmorphism with large anterior fontanel, broad forehead, hypertelorism, downslanting palpebral fissures, low-set and/or malformed ears, abnormal palate, micrognathia and/or retrognathia, pegged teeth, abnormal palmar creases, broad thumbs, cardiovascular abnormalities, skeletal abnormalities, joint dislocations and/or contractures, and undescended testes [1, 2, 4, 7, 8].

Recently, translocations in the 7p22 region were proposed as a candidate for autism [9]. A case of a boy diagnosed with autism, no dysmorphic features, and a de novo balanced translocation 46, XY,t(7;16)(p22.1;p11.2) suggests that overexpression of gene* RNF216* (localized to 7p22.1 by the Mammalian Gene Collection) resulting in abnormalities in E3 ubiquitin ligase may be linked to autism as well as other developmental and psychiatric conditions [9, 10].

We report a 29-month-old patient, recently diagnosed by his pediatrician with autism spectrum disorder, who was sent for genetic evaluation. He was found to have significant speech delay, poor eye contact, and several facial anomalies including brachycephaly and prominent ears. Whole genome microarray demonstrated a 1.3 Mb interstitial duplication of 7p22.1, the second smallest interstitial 7p duplication reported in the literature to date.

## 2. Clinical Report

The patient was a 29-month-old Hispanic male, referred for evaluation of developmental delay. The patient was born at 39 weeks gestation by normal spontaneous vaginal delivery after an uncomplicated pregnancy; birth weight was 3.528 (51–75th centile), head circumference was 34.3 cm (26–50th centile), and length was 52.1 cm (51–75th centile). At birth physical exam he was noted to have wide-spaced eyes, febrile, coarse, and decreased breath sounds, tachypnea, subcostal retractions, umbilical hernia, right undescended testes, and rocker bottom feet as per medical record. The patient was transferred to neonatal intensive care unit (NICU) for progressive respiratory distress and suspected sepsis and was placed on high flow nasal cannula and antibiotic therapy. Karyotype done 46XY. Patient has no siblings and parents were nonconsanguineous. Family history was remarkable for maternal grandmother having three miscarriages.

Echocardiogram at birth showed a large patent ductus arteriosus (PDA) (4 mm) with left to right shunt, mild tricuspid regurgitation (PG 33 mmHg), and patent foramen ovale (3 mm) with left to right shunt, no coarctation of the aorta, otherwise normal. Repeat echocardiogram on day 18 of life showed no PDA and showed mild tricuspid regurgitation (PSG 29 mHg) revealing mildly elevated pulmonary systolic pressure, otherwise normal. Other testing in medical record consist of X-ray of right foot with no congenital abnormality appreciated, unremarkable renal ultrasound, head ultrasound negative for IVH and testicular US that showed right testicle located at right external inguinal ring.

The review of systems was positive for brachycephaly, no eye contact, rolling his head side to side before going to sleep, unilateral right cryptorchidism, feet deformity which resolved spontaneously, and developmental delay. The patient walked at 16 months of age and did not use any words and did not point for what he wanted. Though diagnosed with ASD, no typical ritualistic behaviors were described. Despite not being able to speak, he attempted to communicate with family.

**Figure 1:**
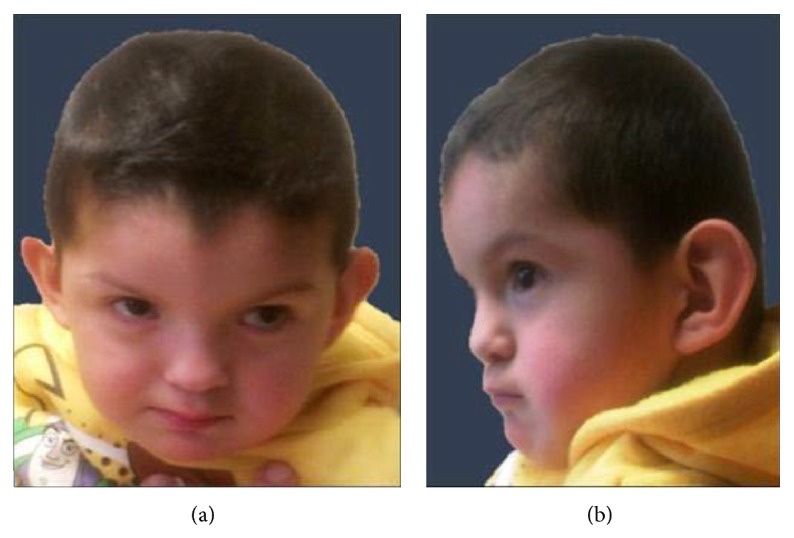
Phenotypic facial features of our patient at the first evaluation in the Driscoll Children's Hospital McAllen Genetics Clinic at 29 months of age. Notable findings include brachycephaly, inner canthal distance of 31 mm (90th centile for age), and prominent lower face and right ear protruded more than left ear.

**Figure 2:**
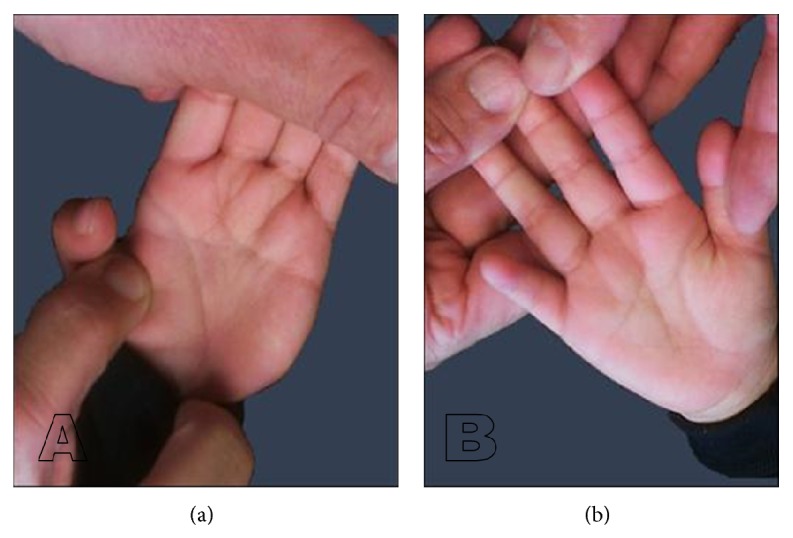
Palmar features. (a) The left palmar creases bridged to form one and distal extends to 2-3 interspace. (b) The right distal palmar crease extends to the 2-3 interspace.

On physical exam, weight was 15.8 kg (90–95th centile) and OFC was 49 cm (50th centile). The head was brachycephalic and the anterior fontanel was closed. Hair was straight and black and of normal distribution and density. There were two posterior whorls and bifrontal upsweeps with a widow's peak. The palpebral fissures were horizontal, inner canthal distance was 31 mm (90th centile), and lower face was prominent. Nasal width was 31 mm (90–95th centile). His mouth was 50 mm (90–95th centile) wide with normal vermillion. Both ears measured 62 mm (90–95th centile), the right ear protruded more than the left ear, and both have a flat posterior helix (Figure 1). Right testicle was not palpable in scrotum. The right distal palmar crease extends to the 2-3 interspace with a small bridged proximal crease. The left palmar creases bridged to form one (Figure 2). There was dorsally placed second toes and flat arches; the toenails were convex. The patient cooperated poorly with examiner and muscle tone was difficult to assess.

**Figure 3:**
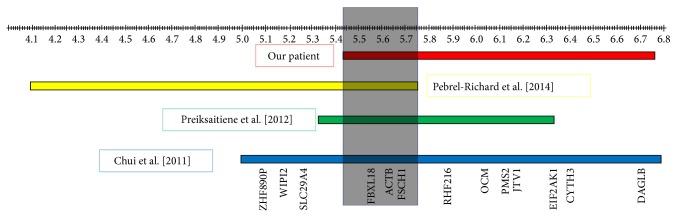
Graphic representation of chromosome 7 with array CGH results Arr 7p22.1 (5,436,367–6,762,394) × 3 in our patient as well as in the patients reported by Chui et al. [4], Preiksaitiene et al. [1], and Pebrel-Richard et al. [11].

Genetic testing included a fragile X PCR DNA analysis, with 31 CGG repeats. Whole genome chromosome SNP microarray (REVEAL) analysis showed a 1.326 Mb interstitial duplication of 7p22.1 >p22.1 arr 7p22.1 (5,436,367–6,762,394) × 3. This interval includes 14 OMIM annotated genes (*FBXL18, ACTB, FSCN1, RNF216, OCM, EIF2 K1, AIMP2, PMS2, CYTH3, RAC1, DAGLB, KDELR2, GRID2IP, and ZNF12*) (Figure 3). Test was interpreted as “possible familial variant” per report.

A duplication variant at Xp22.31 (6,455,151 to 8,135,644) × 2 was also detected. Although deletion of this region spanning the STS gene is associated with ichthyosis in males, familial passage of duplications of this region to normal males has been well documented. Females are unaffected by either deletion or duplication. No extended contiguous regions of homozygotic alleles associated with UPD (single chromosome) or consanguinity (multiple chromosomes) were observed.

Because both anomalies were considered “normal variants,” parental samples were not able to be studied due to healthcare insurance refusing to cover genetic testing at this time.

The patient continued to follow up in the Genetic Clinics at Driscoll Children's Hospital. He was subsequently placed on Guanfacine by his pediatrician for aggressive behavior and outbursts of screaming and walking out of the house during a tantrum. During his last visit in 2013, the patient was 3 years and 9 months old and had a 6-single-word vocabulary. Though still diagnosed with ASD, no typical ritualistic behavior was described by parents and despite his speech delay, he attempted to communicate with family through gestures. During examination, he would at times establish eye contact and share his toy truck with examiner.

## 3. Discussion

Chui et al. [4] reported a case of a 28-month-old Hispanic male with features of a 7p21 duplication syndrome that included developmental and speech delay and craniofacial abnormalities similar to our patient, such as prominent forehead and hypertelorism, as well as cryptorchidism and bridged palmar creases; other abnormalities not seen in our patient included anteverted nares and anterior fontanel closure delay. The duplication was 1.7 Mb in size and located at 7p22.1 region (arr7 p22.1 (5,092,748–6797,449) × 3 (hg18)). Preiksaitiene et al. [1] reported a case of a 14.5-year-old female with a smaller size duplication, a 979.8 Kb located at 7p22.1 region (position 5,337,072–6,316,915 × 3), who had developmental and speech delay, low-set and protruding ears, slanting down palpebral fissures, ocular hypertelorism, midface hypoplasia, microretrognathia, tapering fingers, abnormal palmar dermatoglyphic patterns, and short 5th toes. In both cases, it was found that the duplication was absent in the parents and therefore occurred* de novo*. The duplication in our patient does overlap completely with the patient reported by Chui et al. [4] and partially with the patient reported by Preiksaitiene et al. [1].

A more recent article by Pebrel-Richard et al. [11] presented a 3-year-old boy with a 1,559 Mb microduplication (4,207,513 Mb–5,766,245 Mb) located at the 7p22.2p22.1 region. Their patient presented with psychomotor developmental delay and unusual facial features. He had expressive and receptive language impairment. Physical examination showed prominent forehead, widely spaced eyes, high-arched eyebrows, downslanted palpebral fissures, anteverted nares, large mouth with thin vermilion, and low-set and small ears with narrow external auditory canals, as well as undescended testes, joint hypermobility, and flat arches of feet [11]. Authors attempt to refine a critical region by describing a 430 Kb region of overlap between their patient and Bousman et al. [12]. Our patient further refines this section to 330 Kb region, between 5,436,367 and 5,766,245, which encompasses four RefSeq genes:* FBXL18, ACTB, FSCN1, and RNF216*, where only* RNF216* (OMIM 6609948) and* ACTB* (OMIM 102630) are known to cause diseases in humans (Figure 3).

Papadopoulou et al. [2] and Zahed et al. [3] presented a list of abnormalities described in the literature as an attempt to establish a phenotype or clinical spectrum in patients with 7p duplication. Among these abnormalities, there were described craniofacial dysmorphism, brachycephaly, macrognathia, cryptorchid testes, mental retardation, and one case of autism. Our patient's previous medical records did not include information regarding delayed closure/large fontanels, often described as a common physical finding in reported cases of 7p duplications. When comparing the cases described by Chui et al., Preiksaitiene et al., and Pebrel-Richard et al. with ours, our patient presented many significant similarities but only some of the craniofacial dysmorphic features (Table 1), even though a significant overlap of genes exists when compared to their reported cases, including the* ACTB* gene which has been proposed as a strong candidate gene for the alteration of craniofacial development [1]. This could be due to incomplete penetrance and/or variable expressivity of microduplications/deletions of the same region with resulting different clinical phenotypes [11].

Due to microarray testing of patients with intellectual disability and/or congenital anomalies becoming readily available, there are stronger links between 7p microduplications and developmental disorders, such as autism, speech delay, and mental retardation. The role of other genes in this region such as* RNF216L (Q6NUR6),* which encodes an E3 ubiquitin-protein ligase and is expressed in a variety of human tissues (brain) at all developmental stages [9], is associated with protein quality control as well as regulation of transcription factors such as p53 and androgen receptors [12]. Ubiquitin-ligase complexes have been linked to a number of psychiatric diseases such as bipolar disorder and schizophrenia, as well as developmental disorders including autism (with higher blood levels of E3 ubiquitin in comparison to controls), intellectual disability, Angelman syndrome, and recessive juvenile Parkinson's disease [9]. The* ACTB* gene, encoding b-actin, an essential component of the cytoskeleton, as mentioned before has been suggested as a candidate gene for craniofacial dysmorphism associated with 7p22.1 duplication.

*FSCN1*, which codes for fascin, a protein involved in nerve growth and development, is expressed in mature dendritic cells, epithelial cells, glia, and neurons and plays a critical role in dendritic cell functions and with the accurate establishment of neuronal circuits [13, 14]. Studies on prenatally stressed rats, characterized by an anxious/depressive phenotype associated with neuroadaptive changes in the hippocampus, showed significant changes in the expression of this protein, which may be related to early life stress triggered developmental programming [15]. Similarly, studies have correlated the reduction in dendritic arborizations with intellectual disability, showing a decreased neuronal size and a major cell packing density in patients with a defined neurological disorder. Dendritic abnormalities could lead to a cognitive deficit by reducing the synaptic density or by arresting the synaptic development [15]. Furthermore, microarray assays revealed a significant downexpression of the FSCN1 gene in CREB binding protein-depleted cells found in Rubinstein–Taybi syndrome that is characterized by intellectual disability and growth restriction, multiple congenital malformations such as broad thumbs and big toes, heart defects, cryptorchidism, and increased tumor risk [16]; some of these features are also present in 7p22 patients.

Further investigation is needed in order to determine the relation between these genes which are poorly understood and the characterization of a 7p22.1 duplication syndrome. Recognition of the clinical spectrum in patients with a smaller duplication of 7p should prove valuable for determining the minimal critical region, helping delineate a better prediction of outcome and genetic counselling in patients with duplications in this region [3].

## Figures and Tables

**Table 1:** Comparison of patients with 7p.22.1 patients^∗^.

	Chui et al. [4]	Preiksaitiene et al. [1]	Pebrel-Richard et al. [11]	Our patient
Duplication region	7p22.1 (5,092,748–6,797,449) 1.7 Mb in size.	7p22.1 (5,337,072–6,316,915) 1 Mb in size.	7p22.1 (4,207,513–5,766,245) 1.5 Mb in size.	7p22.1 (5,436,367–6,762,394) 1.3 Mb in size.

Facial characteristics	Open anterior fontanel (20 mm), frontal bossing with a flat, broad, nasal bridge, anteverted nares, ocular hypertelorism, low-set and posteriorly rotated ears with a left preauricular pit, and wide-spaced and pegged teeth.	Low-set and protruding ears, downslanting palpebral fissures, ocular hypertelorism, short nose, anteverted nares, midface hypoplasia, facial asymmetry, severe microretrognathia, high and narrow palate, microstomia, thin vermillion of the lips, and midline pseudocleft upper lip.	Prominent forehead, widely spaced eyes, high-arched eyebrows, downslanted palpebral fissures, anteverted nares, large mouth with thin vermilion, and low-set and small ears with narrow external auditory canals.	Brachycephaly, hypertelorism, prominent lower face, and right ear protruded more than left ear.

Presence of developmental delay	Speech delay. No intelligible words at 33 months.	Difficulty in walking and spastic diplegic cerebral palsy. No mention of verbal abilities.	Few words at 3 years of age. Expressive language impairment was apparently more severe than was receptive language. Could not jump or run and showed slow execution of movements and drawing difficulties.	Poor eye contact and speech delay. Diagnosed with autism spectrum disorder.

Other malformations	Mild kyphosis, bilateral bridged palmar creases, broad thumbs, and an undescended left testis.	Tapering fingers, abnormal palmar dermatoglyphic patterns, contractures of the Achilles tendons, scoliosis, short 5th toes.	Undescended testes, joint hypermobility, and flat arches of feet. Gait was unstable.	Undescended right testicle. The right distal palmar crease extends to the 2-3 interspace with a small bridged proximal crease. The left palmar creases bridged to form one.

Other tests	Normal head UA, normal thyroid function, bone age of left wrist of 16 months at 24 months; echocardiogram: small patent foramen ovale versus a small and hemodynamically insignificant secundum atrial septal defect (ASD); head CT: asymmetry of the anterior fontanel and slight prominence of the right frontal and left occipital bones with no hydrocephalus.	EEG showed diffuse changes in brain electrical activity and increased stimulation in deep brain structures, predominantly in frontal, temporal, and parietal regions. A CT scan of the brain was remarkable for moderate internal hydrocephalus. Electrocardiogram showed signs of vegetodystonia.	Cranial MRI confirmed suspected moderate hydrocephalus and showed a small corpus callosum. Echoencephalogram showed no abnormalities. Ophthalmic examination identified hypermetropia and astigmatism. Hematological, endocrinology, and metabolic tests were normal. Normal thyroid function.	Echocardiogram at birth: large patent ductus arteriosus (PDA) with left to right shunt, mild tricuspid regurgitation, and patent foramen ovale with left to right shunt. Right testicle located at right external inguinal ring on US. Normal head and renal US.

^∗^In previously reported cases of 7p22.1 duplication has arisen de novo. In our patient, parental testing was not available.
